# Prevalence of four different subgenotypes of genotype 4 hepatitis E virus among swine in the Shanghai area of China

**DOI:** 10.1186/1751-0147-50-12

**Published:** 2008-05-31

**Authors:** Yijia Yan, Wen Zhang, Quan Shen, Li Cui, Xiuguo Hua

**Affiliations:** 1 Shanghai Key laboratory of Veterinary Biotechnology, School of Agriculture and Biology, Shanghai JiaoTong University, 800 Dongchuan Road, Shanghai 200240, PR China

## Abstract

**Background:**

Hepatitis E virus (HEV) is a zoonotic pathogen of which swine was reported as major reservoirs. HEV has been divided into 4 different genotypes according to phylogenetic analysis. Recent reports showed that genotype 4 HEV is freely transmitted between humans and swine in eastern China, including Shanghai area. This paper investigated the recent infection status of HEV among swine population of Shanghai area in China.

**Methods:**

480 swine faecal specimens were collected from 23 farms which distribute all over Shanghai from September to November, 2007 and tested for the presence of HEV RNA by the polymerase chain reaction (PCR).

**Results:**

Our results showed that 26.1% (6/23) of the swine farms were positive for HEV RNA and the positive rate of the six farms were ranged from 9.1% to 33.3%. The HEV RNA positive rate for total samples were 5% (24/480). The resulted positive band specific for HEV was sequenced and sequence analysis indicated that all of these isolates belonged to genotype 4 HEV. Phylogenetic analysis showed that the 24 isolates clustered into 4 distinct subgroups, sharing 83.3–89.7% inter-subgroup and 97–99% intra-subgroup identities. More over, isolates in three of the four subgroups closely clustered with previous identified strains, sharing up high to 97% identity with them.

**Conclusion:**

These results suggested that there were 4 different subgenotypes of HEV prevalent in Shanghai, and some of them may not be indigenous to Shanghai but introduced from other geographic regions.

## Background

Hepatitis E virus (HEV), a member of the genus Hepevirus, is a non- enveloped virus with a positives- stranded RNA genome approximately7.2 kb in length [1]. HEV has been proven to transmit by the faecal- oral route, and outbreaks of hepatitis E are attributed to water contaminated with HEV. HEV and antibodies to HEV have been reportedly found in a wide variety of animals, especially swine [2-5]. A hypothesis has arisen that zoonosis is involved in the transmission of HEV, especially for the cases in non- endemic areas. Recently, more direct evidences for zoonotic HEV transmission were reported [6]

The first animal strain of HEV, designated swine HEV, to be isolated and characterized was obtained from a pig in the United States [7]. Subsequently, many HEV samples from swine in over a dozen countries have been identified. HEV isolates were divided into four distinct genotypes according to sequence and phylogenetic analyses. Genotype 1 was previously believed to only infect humans, but reportedly detected from a pig in Cambodia recently [8]. Genotype 2 has only been identified in humans in Mexico and Africa (Nigeria, Chad). Genotype 3 is prevalent in swine herds and humans all over the world and Chinese genotype 3 HEV was first discovered in eastern China in 2006 [9]. Genotype 4 HEV was first detected in humans in 1993 [10] and is mainly distributed in China, Japan, India, Indonesia, and Vietnam. Genotype 4 HEV has a wide host range, being prevalent in humans, swine, and some other animals. These four genotypes of virus are thought to comprise a single serotype [11]. Hepatitis E was first recognised in China after a large epidemic in the south part of Xinjiang Uighur autonomous region [12]. Since 2000, Genotype 4 HEV has become the dominant cause of hepatitis E disease in China [13]. A recent report showed that genotype 4 HEV is freely transmitted between humans and swine in eastern China [14]. In the present study, we tested the 480 faecal samples collected from 23 commercial pig farms in Shanghai area of China for HEV RNA in order to investigate the current status of HEV prevalence in the swine population.

## Materials and methods

### Samples

**Figure 1:**
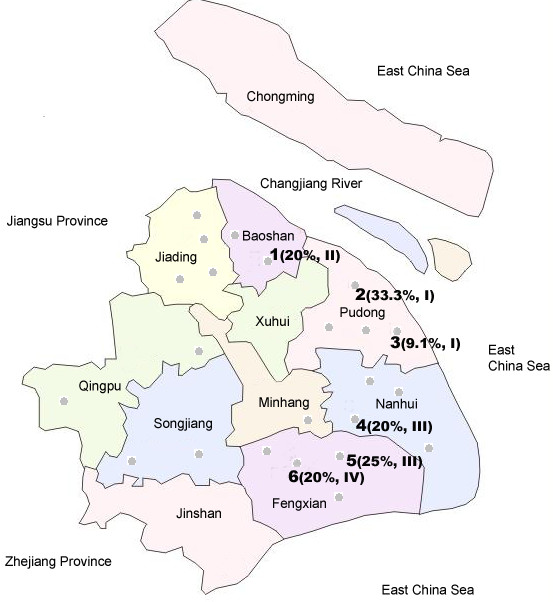
The maps of Shanghai of China. Black points indicate the sites of 23 farms we sampled from. "1", "2", "3", "4", "5" and "6" show six HEV positive farms; the HEV positive rate and subgroup that the HEV strains belong to are indicated in the bracket.

480 swine faecal specimens were obtained from 23 swine farms in swine- farming districts of Shanghai city in China from September to November, 2007. The sampling sites were marked as black point in figure 1, a map of Shanghai. All the swine sampled from were 2–4 months old. Specimens were carefully collected to avoid any contamination. All the samples were converted to 10% (w/v) suspensions in PBS (0.01 M, pH 7.2–7.4) immediately following the sampling. These samples were shipped, frozen, to our laboratory and stored at – 80°C prior to analysis.

### Nucleic acid extraction and designing of PCR primers

Faecal sample suspensions were clarified by centrifugation at 5000 g for 45 min, and 100 μl aliquot of the clarified material was used for viral RNA extraction. Total RNA was extracted by using TRIzol reagent (Invitrogen, USA) in accordance with the manufacturer's protocol. The RNA was finally dissolved in 20 μl RNase- free water. The primers used for HEV sequence amplification in this study were those previously described in reference [15]. The primers were HEV1 [forward primer; 5'-AATTATGCC(T)CAGTAC(T)CGG(A)GTTG-3'] and HEV2 [reverse primer; 5'-CCCTTA(G)TCC(T)TGCTGA(C)GCATTCTC-3'] for the first round of PCR and HEV3 [forward primer; 5'-GTT(A)ATGCTT(C)TGCATA(T)CATGGCT-3'] and HEV4 [reverse primer; 5'-AGCCGACGAAATCAATTCTGTC-3'] for the second round. This set of primers was designed to produce a 348- nt segment of open reading frame (ORF) 2, and were capable of detecting all four known HEV genotypes. Letters in parentheses indicate degenerate bases.

### RT- PCR and nested PCR

Reverse transcriptase PCR (RT- PCR) was performed by using TaKaRa RNA PCR kit (TaKaRa, Japan) according with the manufacturer's instructions. Briefly, 4 μl of RNA solution was analyzed by RT-PCR, plus 2 μl 25 mM Mg2+,1 μl 10 × RT buffer, 1 μl 10 mM (each) dNTP, 20 pmol reverse primer (primer HEV4), 10 U of RNase inhibitor, and 2.5 U of AMV Reverse Transcriptase XL in a total volume of 10 μl. After incubation for 30 min at 42°C, the mixture was incubated for 5 min at 99°C to denature the products and then chilled on ice. 10 μl of the resulting cDNA was amplified by the universal RT- PCR assay using PerfectShot Taq (Loading dye Mix) DNA polymerase (TaKaRa, Japan). Briefly, RT- PCR was conducted in a total volume of 50 μl, containing 10 ul cDNA solution, 25 ul Loading dye Mix and 25 pmol both sense primer and anti-sense primer. The PCR parameters for the first- round PCR included a denaturation step at 95°C for 9 min, followed by 39 cycles of denaturation for 1 min at 94°C, annealing for 1 min at 42°C, extension for 2 min at 72°C, and a final incubation at 72°C for 7 min. 10 ul products of first-round PCR were used as template for the second-round PCR. The parameters for the second- round PCR were similar to the first-round.

### Nucleotide sequencing

The nested PCR products were analyzed in a 1.5% agarose gel containing 0.5 ug/mL ethidium bromide. The expected DNA band specific for the HEV was excised from the gel, purified with the AxyPrep DNA Gel Extraction kit (Axygen, USA) and cloned into pMD T- Vector (TaKaRa, Japan). Both strands of the inserted DNA amplicons were sequenced in a DNA analyzer (Applied Biosystems 3730 DNA Analyzer; Invitrogen, USA).

Standard precautions were used for all procedures to reduce the possibility of sample contamination by amplified DNA molecules. A faecal specimen from a pig infected with swine genotype 4 HEV was used as positive control. PBS instead of faecal suspension, and water instead of RNA samples or cDNA template were used as negative controls in the nucleic acid extraction and RT-PCR, respectively.

### Phylogenetic analysis

**Figure 2:**
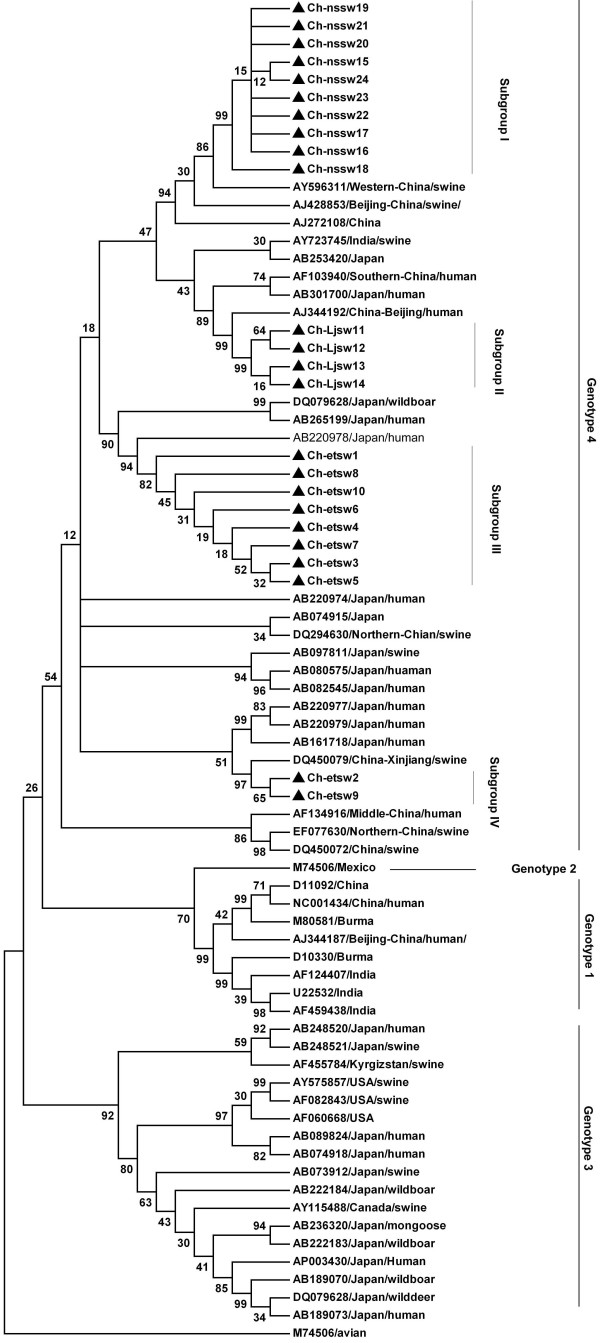
Phylogenetic tree constructed by alignment of the348- nt nucleotide sequence of ORF2 from isolates in this study and references of other animal and human HEV isolates, using the neighbor- joining method and evaluated using the interior branch test method with Mega 4 software. Percent bootstrap support is indicated at each node. GenBank accession no., source and country of origin are indicated. The isolates in this study were marked with solid triangle. Avian HEV strain is included as outgroup.

Sequences were aligned using ClustaX v1.8 or MegAlign program in the DNASTAR software package. Phylogenetic analysis was constructed using the Mega 4 software [16]. Prototype HEV strains used as references in the analysis and their GenBank accession numbers are showed in figure 2. The sequences determined in current study were deposited in GenBank, isolate names were indicated in figure 2 and the accession numbers are EU375319–EU375342.

## Result

### Occurrence of HEV

Table 1 shows the results of detection of HEV-RNA in faecal samples from HEV positive farms. Six out of 23(26.1%) farms from which we sampled were tested positive for HEV. Among the 6 farms, the HEV positive rate of faecal samples ranged from 9.1% to 33.3%. Figure 1 shows the six sites of the HEV positive farms (marked with Arabic numeral 1–6) and the HEV positive rate of each farm (indicated in the bracket). The overall HEV positive rate for all the samples was 5% (24/480).

**Table 1:** Detection of HEV-RNA in faecal samples from HEV positive farms.

No. of farm	Positive samples/Total analyzed (%)	Subgroup of the strains
1	4/20(20%)	II
2	8/24(33.3%)	I
3	2/22(9.1%)	I
4	3/15(20%)	III
5	5/20(25%)	III
6	2/10(20%)	IV

### Phylogenetic analysis

**Figure 3:**
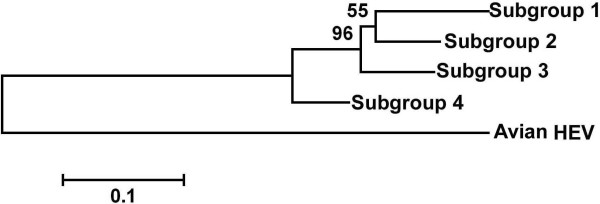
Phylogenetic tree constructed by alignment of consensus sequence of the isolates in each subgroup, using the neighbor- joining method and evaluated using the interior branch test method with Mega 4 software. Percent bootstrap support is indicated at each node. Avian HEV strain is included as outgroup.

The PCR- amplified products of 24 isolates were sequenced. Phylogenetic analyses were conducted using the sequence alignments of these isolates and the references. Figure 1 is the phylogenetic tree constructed based on the 24 isolates in this study and the references by using Mega 4.0 software. Result showed that the isolates we determined all belonged to genotype 4 HEV and they cluster into four different subgroups. The four subgroups clustered closely with previously identified isolates: AY596311 (Xinjiang, Western China), AJ344192 (Beijing, Northern China), AB220978 (Japan), and DQ450079 (Xinjiang, Western China), respectively. Figure 3 is the phylogenetic tree constructed based on the consensus sequences of strains in each subgroup. From the phylogenetic tree, we can see that the phylogenetic relation among the 4 subgroups showed in figure 3 was identical to that of figure 2.

### Divergence and percent identity

The identity among isolates we determined in each subgroup and their closely related strains referenced from GenBank were analyzed. Result showed that intra-subgroup identities of the four subgroups were ranged from 97% to 99%. The identities between the four subgroups and the closely clustered previous strains were 92% (subgroup1 to AY596311), 97% (subgroup 2 to AJ344192), 92–97% (subgroup 3 to AB220978), and 97% (subgroup 4 to DQ450079), respectively. The source sites of the 4 subgroups are shown in figure 1(marked with Roman number in bracket). We can see that subgroup I contains the strains identified in farm 2 and 3, and subgroup III includes the strains found in farm 4 and 5, and the strains in each of the two subgroups have high nucleotide sequence homology (97–99%), suggesting these strains from each two farms were from a common source of infection. Table 2 showed the percent identity and divergence among the 4 subgroups. The identity and divergence among the four subgroups were ranged from 83.3–89.7% and 12.4–19.6, respectively. The overall mean distance for the 4 subgroups was 0.159.

**Table 2:** The divergence and percent identity between the 4 subgroups (subgroup I–IV) identified in the present study.

Percent Identity			
I	89.7	87.0	83.3
13.7	II	88.8	85.8
14.7	12.4	III	85.8
19.6	16.3	16.2	IV
Divergence			

### Sequence alignment

The consensus nucleotide sequence of each subgroup and the coding amino-acid sequences were aligned using DNAstar software. The results indicated that though the 4 subgroups showed high diversity on the nucleotide level (22.7% of the nucleotide sites have base substitution), they were very conservative on the amino-acid level, only having two sites (1.8%) of amino-acid replacement. Because these sequences are part of the ORF2 region encoding a capsid protein of HEV virions, the conservation probably contributes to the same serotype of these strains.

## Discussion

Accumulated evidence indicates that HEV infection is a zoonosis which involves various animal reservoirs. Pigs stand out as being an animal group with the highest positive rate for HEV RNA, where the isolates were shown to be closely associated with those from humans [17-20]. Among the 4 distinct genotypes, genotype 4 has been dominant in China [13]. Recently, genotype 4 HEV was reported to be widely distributed in humans and swine in Eastern China [14], including Shanghai. In the present study, 24 genotype 4 HEV isolates were detected in 480 pigs in Shanghai of China, which were phylogenetically divided into four different subgroups, and the isolates in subgroup 2, and subgroup 3 shared 97% and 92–97% identity with two human isolates, AJ344192 and AB220978, respectively. The two human isolates were from Beijing of China and Japan, respectively [21,22], which are geographically significantly far from Shanghai. Therefore, we speculated that this may owe to the traveling of humans who were infected with these virus, or trading pigs which carrying these virus.

Swine HEV isolates identified from the same geographic region tended to cluster together [23], and this point was confirmed by some recent studies [24,25]. In the present study, the determined 24 isolates were from 6 different farms in the same geographic region, and these strains phylogenetically clustered into genotype 4 group and formed 4 different subgroups, sharing 83.3–89.7% inter-subgroup and 98–99% intra-subgroup identity. This rather low inter-subgroup nucleotide sequence homology among the strains suggested that these Shanghai swine HEV strains represents four distinct variants among the genotype 4 isolates and the HEV prevalence in Shanghai swine is complicated. Phylogenetic analysis also showed that three of the 4 subgroups had closely clustered with three previously identified strains isolated in different regions even countries, sharing up high to 97% identity with them. This suggested that some the HEV strains prevalent in Shanghai swine populations were not Shanghai-indigenous.

Shanghai lies in the most southern eastern part of China. It is one of the most developed and open cities and has the largest number of trading activities in China. Some evidences indicate that 60% of pigs consumed in Shanghai were imported from other regions of China. Shanghai had history of introducing different species of excellent domestic pigs from other regions of China and even abroad for commercial raising. Therefore, we cannot rule out the possibility of an outside source for some of the isolates in the four different subgroups in the present study: they may spread unnoticedly among pigs and humans through pigs trading or people traveling, as suggested in Taiwan and Japan [26,27]. With the increasing of HEV strains, one can look at reasonably comprehensive collection of international strains for phylogenetic analysis to elucidate the impact of pig trading and humans traveling, and clearly clarify the issues in the future.

Our results suggested the strains that from farm 2 and 3, and that from farm 4 and 5 were from a common source of infection, respectively, sharing relatively high identity (97–99%). Moreover, farm 2 and 4 are near from farm 3 and 5, respectively (figure 1). This may imply HEV can transmit from one farm to the farm nearby through a certain route, such as faecal contamination and people movement, suggesting that the feaces of pig need to be treated under strict conditions and people's movement between pig farms should be well controlled. Although farm 2 and 3 have HEV strains clustered closely in the same subgroup prevalent and are geographically near, the HEV positive rate of them are relatively different (33.3% and 9.1%). This may be caused by non-viral factors, such as sanitary conditions, hosts, facilities, or type of farming.

Although HEV of different genotypes are genetically very heterogenic on nucleotide level, some universal primers were designed which can detect HEV strains from all four known genotypes [15,28]. This set of RT-PCR primers used here belong to one of them and were used in many previously studies. In this study, positive control were added through out experiments and the HEV isolates we determined using this set of primers all belonged to genotype 4 HEV, without genotype 3 isolates. This was contrast to the study results of Ning [9] and our previous data (unpublished), which showed genotype 3 HEV strains existed in Shanghai of China, based on detection of swine serum or faecal specimens collected before 2006. Different sampling site and time may contribute to this point. A definitive conclusion for this issue should be drawn by more research data.

## Conclusion

The genotype 4 HEV isolates prevalent in swine populations in Shanghai area can be divided into 4 subgenotypes and the sequence identities among them ranged from 83.3% to 89.7%. Based on the phylogenetic analysis, the 4 subgenotypes closely clustered with 4 previously identified stains from other different geographic regions, respectively, suggesting that some of the HEV isolates may not be indigenous to Shanghai.

## Competing interests

The authors declare that they have no competing interests.

## Authors' contributions

All authors participated in the planning of the project. XH was the leader of the project. YY and WZ collected all samples and performed the detecting experiments for HEV RNA. WZ and QS done the phylogenetic analysis. All authors read and approved the final manuscript.
